# Achievement of Primary Prevention Cardiometabolic Targets in Women with HIV: An Urgent Call to Action to Pursue Cardiovascular Health

**DOI:** 10.3390/v16040578

**Published:** 2024-04-09

**Authors:** Maria Mazzitelli, Vincenzo Scaglione, Claudia Cozzolino, Marco Zuin, Cristina Putaggio, Beatrice Bragato, Eleonora Vania, Lolita Sasset, Davide Leoni, Vincenzo Baldo, Annamaria Cattelan

**Affiliations:** 1 Infectious and Tropical Diseases Unit, Department of Molecular Medicine, Padua University Hospital, 38128 Padua, Italy; beatrice.bragato@aopd.veneto.it (B.B.); lolita.sasset@aopd.veneto.it (L.S.); annamaria.cattelan@aopd.veneto.it (A.C.); 2 Department of Cardiac, Thoracic, Vascular Sciences, and Public Health, University of Padua, 35131 Padua, Italyclaudia.cozzolino@studenti.unipd.it (C.C.); vincenzo.baldo@unipd.it (V.B.); 3 Department of Translational Medicine, University of Ferrara, 42121 Ferrara, Italy; 4 Infectious and Tropical Diseases Unit, Belluno Hospital, 32100 Belluno, Italy; cristina.putaggio@aulss1.veneto.it

**Keywords:** metabolic risk, cardiovascular risk, women with HIV, primary prevention

## Abstract

Background: Cardiometabolic health has become crucial, especially for women with HIV (WWH). We assessed the achievement of targets for hypertension, dyslipidemia, and diabetes (H/Dy/DT) in primary prevention in a WWH cohort. Methods: Cross-sectional analysis including all WWH in our clinic, excluding those who had a myocardial infarction. H/Dy/DT achievement was assessed by both EACS guidelines and individual cardiovascular risk, CVR (measured by ESC calculator), using logistic regression to evaluate differences in H/Dy/DT achievement between migrant and Italian women. Results: We included 292 WWH, 55.5% Italian and 44.5% migrant women; the median age was 50 (IQR:42–58) years, 94.5% had undetectable HIV-RNA, 55.1% had a high level of education, 27.1% were smokers, and 19.2% did regularly physical exercise. Overall, 76%, 19%, and 5% of women presented a low, a high, and a very high CVR, respectively. Among Italians, 28.4% and 6.2% women presented a high and a very high CVR, respectively. Considering migrants, 7.7% and 3.8% women presented a high and a very high CVR, respectively. Overall, among migrant women, those with a high CVR were more likely to be not at target than those with a low risk (especially for LDL-c and blood pressure among people on treatment), despite the fact that we did not detect a statistically significant difference. By contrast, migrants were more likely to achieve glycemic targets than Italians (*p* = 0.032). Conclusions: H/Dy/DT target achievement is suboptimal, especially in migrants. A more aggressive pharmacological treatment, also assessing adherence to medical prescriptions, and promotion of healthy lifestyle should be urgently implemented, possibly redrawing the current model of care.

## 1. Introduction

For years, the main goal of HIV care was to achieve and maintain HIV-RNA undetectability. Over the last decades, the viral replication became manageable and, thanks to increasingly powerful, effective, and tolerated antiretrovirals in triple or dual therapy, the population living with HIV (PLWH) is living longer, with an increasing life expectancy comparable to that of the general population [1,2]. Furthermore, the prevalence of elderly PLWH is rising worldwide, leading to an increase in the prevalence of comorbidities [3]. Specifically, the most prevalent ones are cardiovascular diseases, cancers, diabetes, dyslipidemia, chronic renal disease, hepatitis B, and hepatitis C [4]. The comorbidity number generally increases with HIV disease severity, but their etiology remains multifactorial (antiretroviral toxicity, aging, inflammation, etc.) [5]. Additionally, the use of several drugs leads to drug interactions and possible toxicities [6,7,8,9,10,11]. This clinical complexity often requires a multidimensional approach and dedicated services, which have been implemented in different settings [6,8,12]. In this scenario, cardiometabolic diseases became the main cause of death, followed by non-AIDS related cancers [13,14,15].

The PLWH faces disparities in the quality of cardiovascular care compared to the population without and is less likely to be prescribed statins and aspirin when needed [16]. Therefore, clinicians turned attention to understanding and identifying the specific cardiovascular risk (CVR) factors and outcomes among different PLWH, including women, who represent about the 50% of the whole population with HIV globally [17]. Moreover, women with HIV (WWH) face unique challenges and vulnerabilities, which can influence their CVR profile [18]. In addition, some gender-related factors such as hormonal changes, pregnancy, and experiences of violence and trauma may interact with the biological processes associated with HIV, increasing CVR [18,19]. Furthermore, PLWH may be more prone to certain traditional CVR factors, such as smoking, substance abuse, sleep disturbances, and mental health disorders, due to the social and structural barriers they face [7,20,21]. Both in WWH and without HIV, the CVR increases after menopause, mostly for the cessation of the estrogen protective effect [22]. However, WWH have a higher prevalence of hypertension, dyslipidemia, obesity, and insulin resistance, which contribute to the development and progression of CVD [18,23,24]. One crucial aspect of studying CVR in women is the recognition of the unique biological, psychosocial, and behavioral factors that contribute to their increased risk [18,25,26,27].

Therefore, understanding the complex interplay between HIV, hormonal factors, and CVD risk in women remains essential to promote tailored preventive interventions reducing the burden of CVDs in this fragile population. The aim of our study was to assess achievement of cardiovascular and metabolic targets in a population of WWH, and secondly, to assess whether achievement of these targets was different between Italian and migrant women.

## 2. Materials and Methods

This is a cross-sectional analysis including all WWH in active follow-up at the HIV outpatient’s unit of the Infectious Diseases Department of Padua University Hospital. This study was conducted according to the principles of good clinical practice and the Declaration of Helsinki. The study protocol was submitted to and approved by the local ethics committee and all women participating provided informed consent (approved on 21 October 2021). From medical health records, we retrospectively collected demographics, clinical information, prevalence of comorbidities, comedications (in particular, statins, anti-hypertensive agents, and hypoglycemic drugs), and laboratory test results (HIV-RNA, CD4+ T cell count, total cholesterol, HDL, LDL, triglycerides, glucose, glycosylate hemoglobin). We also collected anthropometric measures such as body weight, height, waist circumference, and body mass index (BMI). Diagnosis of metabolic syndrome (MetS) was done accordingly to NCEP ATP III criteria [28]. We collected also behavioral information such as: cigarette smoking, alcohol consumption, and physical exercise (all defined as per internationally recognized WHO definitions).

Then we assessed individual achievement of different targets for blood pressure (BP), glycemic parameters, and LDL cholesterol (LDL-c), according to EACS Guidelines, overall and after stratification for CVR and the primary prevention strategies [29]. Due to the study design, women with a previous history of major cardiovascular events (i.e., myocardial infarction and/stroke) were excluded from the analysis. The prediction of 10-year risk of first-onset CVD was calculated using the SCORE2 and SCORE2-OP (for patients aged ≥ 70 years) algorithms, as currently suggested by the European Society of Cardiology (ESC) [30]. More precisely, SCORE2 and SCORE2-OP were calculated using age, smoking status, systolic blood pressure, total cholesterol, HDL, and LDL cholesterol. Risk classes were defined as low to moderate, high, and very high risk [30].

Descriptive statistics were obtained representing categorical variables as frequencies and proportions (%) and summarizing continuous numerical variables with means, medians, and interquartile ranges (IQR). Differences among Italian and migrant women in clinical-sociodemographic characteristics, in target attainment, and in positivity to NCEP ATP III criteria for MetS diagnosis were assessed with the Chi-squared test, or alternatively, the Fisher’s exact test in the presence of an expected frequency lower than five; and with the two-sample *t*-test, or alternatively, the Mann–Whitney U test when normality assumptions were not met. Lastly, multivariable analysis by logistic regression was conducted to investigate whether ethnicity (Italian vs. migrant) or any of the other available variables were possibly associated with protective or risk factors for achieving each of the three targets, BP, glycemic, and LDL-c. Odds ratios (OR), with relative 95% confidence interval (CI) were estimated modelling nonattainment of target as outcome. Results were deemed statistically significant at *p* < 0.05. All data analyses and visualization were conducted in R software (version 4.0.4). and in Python 3.8.18.

## 3. Results

From our cohort of 300 WWH, eight women were excluded due to a history of myocardial infarction. As a result, we included 292 WLWH with a median age of 50 (42.0–58.0) years. Regarding HIV infection, 94.5% had an undetectable HIV RNA (i.e., <50 copies/mL), with a median CD4+ T cell count of 617.5 (448.2–825.2) cell/mm^3^. Overall, 55.1% of women had a high level of education, 27.1% were active smokers, and 19.2% were engaged in regular physical exercise. The median age of women living with HIV and the median age of women on antiretroviral therapy were 16 (IQR: 9–25.8) and 15 (IQR: 8–23) years, respectively, while 16.8% women had a past medical history of AIDS-defining illnesses. Regarding antiretroviral treatment, most of the women were on a triple regimen including either 2 nucleoside reverse transcriptase inhibitors (NRTI) plus an integrase inhibitor (INI) (28.1) or 2NRTI plus a non-nucleoside reverse transcriptase inhibitor (NNRTIs) (27.1%), while 20.5% women were on a combination including an INI plus a boosted protease inhibitor (PI). Overall, 64.4% and 15.6% of women presented multimorbidity and polypharmacy, respectively. The most common comorbidities were dyslipidemia (28.1%) followed by hypertension (27.7%) and anxiety (24%). Overall, 162 (55.5%) women were Italian and 130 (44.5%) were migrant. Italians were older (*p* < 0.0001) and had a higher-level education (*p* = 0.0005), a longer history of HIV infection (*p* < 0.0001), and a higher CD4+ T cell count (*p* = 0.005), compared to migrants. Italians were more likely to have a normal BMI, to be more active in terms of physical exercise, to be smokers, and to be affected by HCV coinfection, lipodystrophy, autoimmune disorders, dyslipidemia, and osteoporosis. Migrant women were more likely to be affected by obesity and diabetes (Table 1).

**Figure 1:**
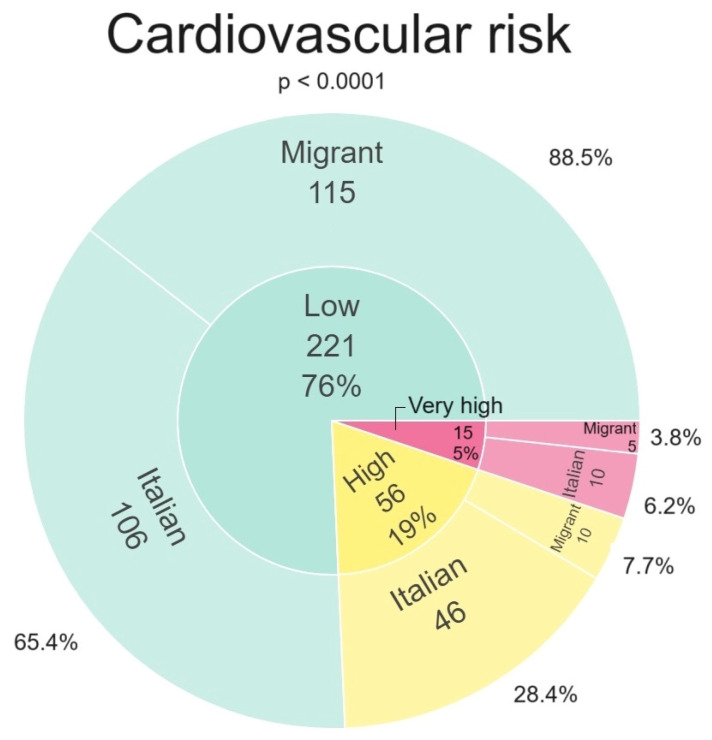
Cardiovascular risk stratification in Italian and migrant women.

In Figure 1 we depict the stratification of the CVR, overall and by the two study groups (migrant women vs. Italian women). Overall, 221/292 (76%) women presented a low to intermediate CVR, 56/292 (19%) presented a high CVR, and 15/292 (5%) presented a very high CVR. Subgroup analyses revealed that 28.4% and 6.2% of Italian women presented a high CVR and a very high CVR, respectively. Considering the migrant group, 7.7% and 3.8% presented a high CVR and a very high CVR, respectively. A statistically significant difference was found between Italian and migrant women groups for both high CVR and very high CVR variables.

**Figure 2:**
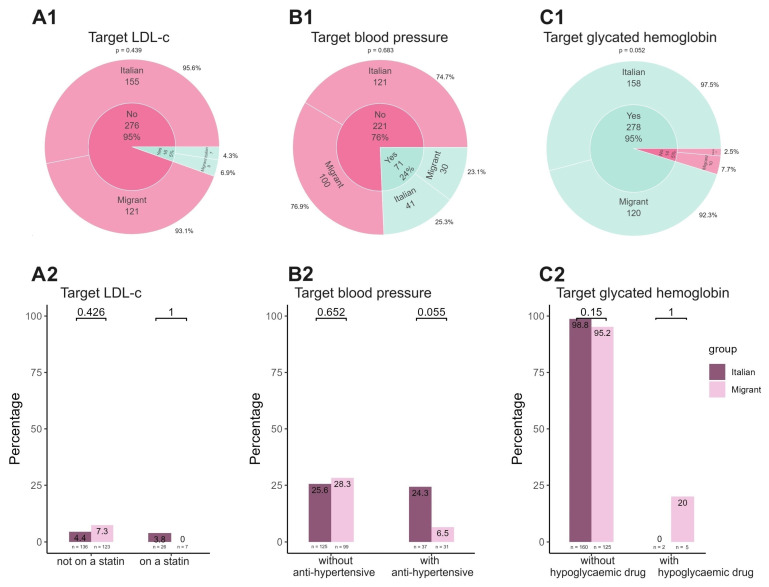
Achievement of each target by both study group and therapeutical interventions. In panel (**A1**–**C2**) we reported the prevalence of achievement of each studied target by considering Italian vs. migrant women and the presence or absence of any treatment for each condition.

Figure 2 summarizes the rate of achievement for each target (LDL-c, A1, blood pressure, B1, and glycated hemoglobin, C1) both for the study group overall and stratified by the presence of cardiovascular and metabolic drugs. Regarding the achievement of LDL-c targets (Figure 2(A1)), overall, 276/292 (95%) women were not meeting the targets. Specifically, among Italians, 155/162 (95.6%) were not on target, while among migrants, 121/130 (93.1%) were not meeting the target. Indeed, only 5% of women were able to reach the target for LDL-c; there were no statistically significant differences observed between Italian women and migrant women in this regard (7/16, 6.9% vs. 9/16, 4.3%, *p* = 0.439). Considering women who were on statins, only 3.8% had an LDL-c on target and none were migrant women (Figure 2(A2)). Considering blood pressure (Figure 2(B1)), overall, 221/292 (76%) did not have values achieving the targets, and 121/162 (74.7%) and 100/130 (76.9%) Italians and migrants were not on target, respectively. Only 24% of women achieved the blood pressure target, with no statistically significant differences between Italian and migrant women (41/71, 25.3% vs. 30/71, 23.1%, *p* = 0.683). Examining the group of women (*n* = 68) undergoing treatment for hypertension (Figure 2(B2)), a greater percentage of Italian women achieved the recommended target blood pressure levels, compared to migrant women, with a trend towards statistical significance (9/37 [24.3%] vs. 2/31 [6.5%], *p* = 0.055).

Conversely, glycemic control demonstrated an opposite trend compared to the two preceding parameters. Indeed, 95% of women overall had a normal glycated hemoglobin (Figure 2(C1)), whereas only 5% did not reach the target, with a statistically significant difference between migrants and Italians (10/14 vs. 4/14, *p* = 0.052). When looking at women receiving hypoglycemic medication treatment (Figure 2(C2)), only 20% of the migrant women and none of the Italian women met the target.

**Figure 3:**
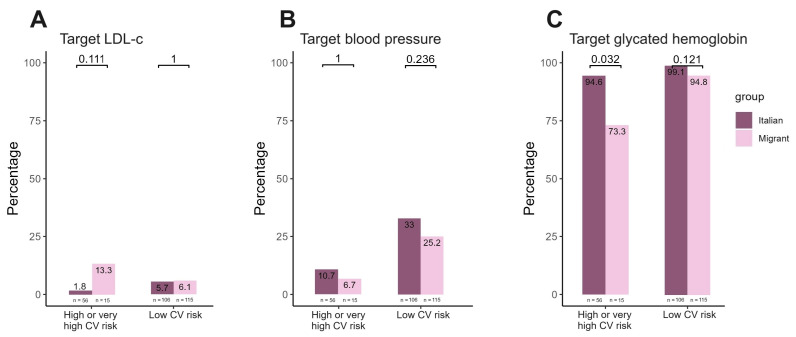
Achievement of target ((**A**) LDL-c; (**B**) blood pressure and (**C**) glycated hemoglobin) by cardiovascular risk stratification.

Figure 3 summarizes target achievement by stratifying the population by CVR. Overall, the group of women with high CVR had a higher likelihood of not meeting the target levels compared to those with low risk, particularly for LDL-c and blood pressure levels (as shown in Figure 3A,B). To this regard, no statistically significant differences were observed comparing the two study groups. In contrast, significant differences were found between migrant and Italian women in terms of achieving glycated hemoglobin targets (Figure 3C) among women with high CVR (*p* = 0.032).

**Figure 4:**
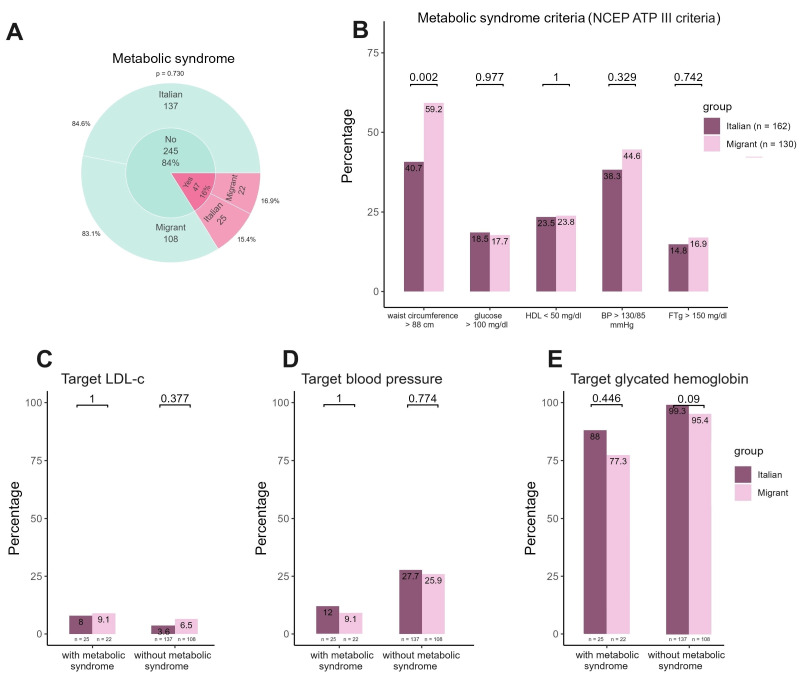
Prevalence of metabolic syndrome, distribution of metabolic syndrome criteria and target achievement in migrant and Italian women with metabolic syndrome. In panel (**A**) we reported the prevalence of metabolic syndrome overall, and by groups (either Italian or migrant women). In panel (**B**) we summarized distribution of each criterion contributing to metabolic syndrome diagnosis, with differences in terms of prevalence between Italian and migrant women. In panel (**C**–**E**) we summarized the rate of achievement for each target in migrant and Italian women with and without metabolic syndrome.

Figure 4 shows the distribution of criteria for the diagnosis of metabolic syndrome overall and for the Italian and migrant women groups separately. Moreover, it depicts the achievement of each target by study group in women with and without metabolic syndrome, respectively. Overall, 47/292 (16%) women presented metabolic syndrome (25 Italian and 22 migrant women, respectively), as shown in Figure 4A. No differences were observed in criteria determining metabolic syndrome between the two groups for glucose, HDL, triglyceride levels, and blood pressure values (Figure 4B). By contrast, when considering waist circumferences greater than 88 cm, migrant women were more likely to present this criterion than Italian ones (*p* = 0.002). When evaluating the attainment of the three distinct targets, it was found that approximately 10% of women, whether they were migrants or Italians, attained the LDL-c targets and blood pressure targets. The attainment of glycated hemoglobin target was achieved by 88% of Italian women and 77.3% of migrant women, with no statistically significant differences observed between the two groups.

Finally, when the regression analysis to assess factors associated with achievement of the three targets was performed, it revealed that age (OR: 1.071, *p* = 0.005) and BMI (OR: 1.157, *p* < 0.001) were factors statistically associated with missed achievement of blood pressure targets, while lipodystrophy for LDL-c (OR: 0.061, *p* = 0.018) and treatment with INI+NRTIs for glycated hemoglobin (OR: 6.4, *p* = 0.048).

## 4. Discussion

CVDs remains the leading cause of death among women worldwide, accounting for 35% of annual female deaths, exceeding the combined mortality rate for all type of cancers [31]. The data is even more significant among WWH, since they face a two-fold increased risk of developing CVD compared to women without HIV [18]. Considering that the life expectancy of WWH continues to rise [2,32], it is of crucial importance to assess and prevent CVDs in this sub-group of PLWH.

In our study population, 24% of WWH presented either a high (19%) or a very high (5%) CVR, with Italian women more significantly represented in both subgroups. In comparison with the 10-year cardiovascular risk (10-CR) assessment of the Italian cohort of female population aged from 35 to 69 years using the CUORE project risk score, our data confirm that WWH have a significantly higher cardiovascular risk: 19% vs. 4.1 for CVR > 10% and 5% vs. 0.4% for risk > 20% [33]. Therefore, it is imperative that the assessment of CVR profile in WWH become a crucial aspect for the development of efficient prevention and care measures. However, our analysis was based on a novel and more accurate stratification strategy using the SCORE2 or the SCORE2-OP algorithms. Indeed, the prediction algorithms used in our analysis provided a sex-specific 10-year risk prediction, calibrated to the mot contemporary and representative CVD rates, and based on the different features of European regions [34]. To this regard, previous investigations assessing the CVR in general population, as well as in PLWH, were derived from non-gender-specific models and not calibrated on contemporary CV estimates [18,35,36]. Frequently, WWH often receive suboptimal CVD-preventive care [16]. Our study demonstrated and quantified the alarming current gaps in both cardiovascular and metabolic prevention in WWH, especially in those at high and very high CVR. Target achievement was insufficient, especially for blood pressure and LDL-c, which are considered the primary drivers and perpetrators of atherosclerosis [37]. Despite the recommendations provided by the EACS guidelines [29], in our study, individual LDL-c treatment targets were achieved by only 5% of women, regardless of whether they were Italian or migrants. Furthermore, a minority of women (33/276, 11.9%), were using statins, achieving the desired LDL-c level in a negligible proportion of cases (3.8%), none being migrant women. Unfortunately, many studies conducted both in the general population and in PLWH, reported suboptimal use and under prescription of statins [38,39,40,41] and achievement of target LDL goals [38,39,40,41,42].

Statins remain underused to due to unfounded concerns about potential interactions with other medications or the development of side effects, as myalgia [43]. Moreover, statin acceptance varies among PLWH [44]. Healthcare providers must consider the potential impact of the nocebo effect and fear when prescribing statins to PLWH, as these factors may contribute to the occurrence of adverse effects and statin discontinuation. In a comprehensive systematic review and meta-analysis including 91,594 PLWH and assessing major outcomes associated with statin treatment, the risk of all-cause mortality, any adverse effects, and the development of diabetes mellitus was significantly lower in the group receiving statins [45]. Additionally, prevalent symptoms in PLWH, including asthenia, elevated liver enzyme levels, increased CPK, and myalgia, were noted, but they were not statistically associated with statin use [45,46,47].

Moreover, the commitment to proper statin prescription in PLWH must become increasingly critical considering the REPRIEVE trial outcomes [48].

In this randomized placebo-controlled trial involving 7500 PLWH with low to moderate CVR, the results revealed a 35% decrease in the risk of mortality from CVD over a median follow-up period of 5.1 years with pitavastatin treatment [48]. It is worth noting that the effect sizes were similar in men and women. This feature is particularly interesting because, among women, immune activation is higher, confirming the role of statins in the reduction in some markers of inflammation, monocyte activation markers, and vascular inflammation [49].

Hypertension plays a crucial role among traditional risk factors in the cardiovascular risk of WWH. In our cohort, almost 28% of women presented a past medical history of hypertension. However, based on blood pressure measurements during clinical check, we detected 76% of women not on BP target according to EACS guidelines [29]. It could be speculated that white coat or masked hypertension could have biased the results, as well as the absence of systematic screening using the 24-h blood pressure Monitoring (ABMP).

Nevertheless, within our study population, a certain balance exists between the two cohorts of women concerning the risk factors associated with hypertension. Italian women, on the one hand, were much older, had a higher smoking rate, and engaged in more physical activity compared to their African counterparts. Conversely, African women displayed a significantly larger waist circumference and higher BMI when compared to Italian women. These results together the target achievement of glycated hemoglobin also explain why there are no significant differences in the prevalence of metabolic syndrome in the two groups of women.

A cause for significant concern is the low achievement of target blood pressure levels among women undergoing antihypertensive treatment. Only 24% of Italian women and 6.5% of migrants have successfully reached the blood pressure goals. These percentages decrease even more among women with very high and high cardiovascular risk, with the figures being 10.7% for Italian women and 6.7% for migrant women, respectively. Similarly, among women receiving hypoglycemic treatment, even though a very small number, the attainment of target glycemic levels is notably low, with only 20% of migrants and none of the Italians reaching the specified glycemic target.

Therefore, understanding WWH CVR profile is essential for effective prevention and treatment. Many studies have found higher rates of CVD risk factors in these populations, but we still know little.

This study is affected by several limitations: the cross-sectional nature with a retrospective data collection, the limited sample size, and the absence of a comparative group both of men and women without HIV. Moreover, the absence of a long-term cardiovascular follow-up, exploring the rates of novel cardiovascular events cannot allow us to estimate the CV disease progression and efficacy of current primary preventive strategies. Furthermore, we did not assess the impact of a possible antiretroviral changes to “more lipid-friendly” regimens on lipids, which could be also an option in an increasing number of PLWH with metabolic issue [50,51]. Moreover, we could have gotten more insight by better assessing some socioeconomic factors, including income, dietary habits, employment status, social support, and mental health, as factors all possibly implicated in cardiometabolic health. Lastly, we do not report a detailed analysis of lipids, hypertension and diabetes medication types, dosages, and adherence which could have provided valuable suggestions into their effectiveness and optimization.

Further research is needed to determine how different ART regimens may affect CVR, and relative outcomes, in women. To fill these gaps in knowledge, large prospective studies must target WWH and measure their CVR. These studies should examine how hormonal factors, societal determinants, and ART affect CVD outcomes. Interventions targeting WWH CVR factors are needed in addition to epidemiological studies. Tailored therapy should address biological, psychological, and behavioral risk factors for CVD and focus on improving healthcare accessibility, mental health, and good habits. All stakeholders, and governments should address the unique challenges and vulnerabilities of WWH to reduce CVD in this specific population.

## 5. Conclusions

In conclusion, women living with HIV encounter a heightened risk of CVD compared to the general population. This increased risk is influenced by a combination of biological, psychosocial, and behavioral factors. It is imperative to urgently address these unmet needs through dedicated research, preventive measures, and effective management strategies to enhance cardiovascular health outcomes for WWH. Recognizing and addressing the distinct challenges and vulnerabilities faced by this population, healthcare providers and policymakers can proactively work towards alleviating the burden of cardiovascular disease in WWH.

## Figures and Tables

**Table 1:** Cohort description and differences between migrant and Italian women.

Characteristics	Overall, *n* = 292	Migrant Women, *n* = 130	Italian Women, *n* = 162	*p*-Value
Age (years), median (IQR)	50 (42.0–58.0)	43.0 (37.0–49.0)	56.0 (50.0–60.0)	<0.0001
Level of education, *n* (%)				0.0005
Degree	20 (6.8)	8 (6.2)	12 (7.4)	
High school diploma	141 (48.3)	39 (30.0)	102 (63.0)	
Middle school diploma	78 (26.7)	33 (25.4)	45 (27.8)	
Primary school	52 (17.8)	49 (37.7)	3 (1.9)	
None	1 (0.3)	1 (0.8)	0 (0)	
Years with HIV, median (IQR)	16.0 (9.0–25.8)	11.5 (6.0–17.0)	24.0 (13.0–30.2)	<0.0001
Nadir CD4+ T cell count (cell/μL), median (IQR)	270.0 (153.0–416.0)	280.0 (153.0–416.0)	263.5 (156.2–419.5)	0.7677
Previous AIDS events, *n* (%)	49 (16.8)	20 (15.4)	29 (17.9)	0.8501
Current CD4+ T cell count (cell/μL), median (IQR)	617.5 (448.2–825.2)	565.0 (430.2–753.5)	670.0 (470.8–904.8)	0.0059
Undetectable viremia (<50 copies/mL)	276 (94.5)	120 (92.3)	156 (96.3)	0.1366
Years on ART, median (IQR)	15 (8–23)	12 (6–17)	20 (10.8–27)	<0.0001
Current ART, *n* (%)				0.0020
2NRTI + INI	82 (28.1)	41 (31.5)	41 (25.3)	
2NRTI + NNRTI	79 (27.1)	43 (33.1)	36 (22.2)	
INI + NRTI	35 (12.0)	19 (14.6)	16 (9.9)	
2NRTI + PI	12 (4.1)	1 (0.8)	11 (6.8)	
INI + PI	60 (20.5)	15 (11.5)	45 (27.8)	
INI + NNRTI	14 (4.8)	6 (4.6)	8 (4.9)	
Other	10 (3.4)	5 (3.8)	5 (3.1)	
HBV (HBsAg), *n* (%)	20 (6.8)	13 (10.0)	7 (4.3)	0.0562
HCV (Ab), *n* (%)	50 (17.1)	4 (3.1)	46 (28.4)	<0.0001
Smoking status, *n* (%)				<0.0001
No	174 (59.6)	103 (79.2)	71 (43.8)	
Yes	79 (27.1)	21 (16.2)	58 (35.8)	
Ex	39 (13.4)	6 (4.6)	33 (20.4)	
Physical activity, yes, *n* (%)	56 (19.2)	15 (11.5)	41 (25.3)	0.0030
Risk factor for HIV acquisition, *n* (%)				<0.0001
Blood products	4 (1.4)	2 (1.5)	2 (1.2)	
Heterosexuals	255 (87.3)	127 (97.7)	128 (79.0)	
IVDU	31 (10.6)	0 (0)	31 (19.1)	
Vertical	2 (0.7)	1 (0.8)	1 (0.6)	
Alcohol abuse, *n* (%)				0.4479
No	273 (93.5)	124 (95.4)	149 (92.0)	
Yes	17 (5.8)	6 (4.6)	11 (6.8)	
Ex	2 (0.7)	0 (0)	2 (1.2)	
BMI, kg/m^2^, median (IQR)	25.1 (22.2–29.3)	27.9 (23.9–31.6)	23.8 (21.5–26.7)	<0.0001
Underweight (BMI < 18.5), *n* (%)	8 (2.7)	3 (2.3)	5 (3.1)	0.7361
Normal weight (BMI 18.5–24.9), *n* (%)	132 (45.2)	39 (30.0)	93 (57.4)	<0.0001
Overweight (BMI 25 to 29.9), *n* (%)	84 (28.8)	41 (31.5)	43 (26.5)	0.3487
Obese (BMI ≥ 30), *n* (%)	68 (23.3)	47 (36.2)	21 (13.0)	<0.0001
Waist circumference, cm, median (IQR)	90.0 (83.0–99.0)	92.5 (85.0–104.0)	87.0 (80.0–95.0)	0.0004
Malignancies, *n* (%)	22 (7.5)	9 (6.9)	13 (8.0)	0.0176
Renal failure, *n* (%)	22 (7.5)	9 (6.9)	13 (8.0)	0.7230
Dyslipidemia, *n* (%)	82 (28.1)	19 (14.6)	63 (38.9)	<0.0001
Lipodystrophy, *n* (%)	25 (8.6)	5 (3.8)	20 (12.3)	0.0099
Hypertension, *n* (%)	81 (27.7)	37 (28.5)	44 (27.2)	0.8051
Cirrhosis, *n* (%)	9 (3.1)	2 (1.5)	7 (4.3)	0.3072
Diabetes mellitus, *n* (%)	14 (4.8)	11 (8.5)	3 (1.9)	0.0086
Osteoporosis, *n* (%)	50 (17.1)	11 (8.5)	39 (24.1)	0.0004
COPD, *n* (%)	15 (5.1)	3 (2.3)	12 (7.4)	0.0498
Anxiety, *n* (%)	70 (24.0)	24 (18.5)	46 (28.4)	0.0481
Depression, *n* (%)	42 (14.4)	13 (10.0)	29 (17.9)	0.0559
Neurological diseases, *n* (%)	31 (10.6)	9 (6.9)	22 (13.6)	0.0665
Autoimmune diseases, *n* (%)	20 (6.8)	4 (3.1)	16 (9.9)	0.0222
N. Comorbidities, median (IQR)	2.0 (1.0–4.0)	2.0 (1.0–3.0)	3.0 (1.0–4.0)	<0.0001
Multimorbidity, *n* (%)	188 (64.4)	71 (54.6)	117 (72.2)	0.0018
n drugs, median (IQR)	2 (1–3)	2 (1–3)	2 (1–4)	0.0149
Polypharmacy, *n* (%)	45 (15.6)	15 (11.5)	30 (18.9)	0.0873

## Data Availability

All data herein showed are available from corresponding author upon reasonable request.
